# Revisiting nitrogen assimilation strategies in the mammalian gut: lessons from *Enterobacteriaceae* as pathobiont models and a challenge to the limitation paradigm

**DOI:** 10.1007/s00203-025-04404-1

**Published:** 2025-07-28

**Authors:** Lisa C. Harling, Aaron L. Hecht, Frank Meyer, Gary D. Wu

**Affiliations:** 1 Division of Gastroenterology and Hepatology, Hospital of the University of Pennsylvania, Philadelphia, PA 19104 U.S.A.; 2 Department of General, Visceral, Vascular and Transplant Surgery, University Clinic Magdeburg, 39120 Magdeburg, Saxony-Anhalt Germany

**Keywords:** Gut microbiome, *Enterobacteriaceae*, Gut nitrogen sources, Nitrogen-limitation, Nitrogen scavenging

## Abstract

The intestinal microbiome is dependent on nitrogen to support growth, yet the extent of nitrogen-limitation for microbial expansion in the mammalian gut remains debated. Classical perspectives describe the colon as nitrogen-limited due to the early absorption of dietary nitrogen in the small intestine, prompting the evolution of nitrogen-scavenging. In this mini-review, we focus on *Enterobacteriaceae*, facultative anaerobes, and clinically relevant pathobionts to examine nitrogen-scavenging and -assimilation systems. Drawing from in vitro, in vivo and ex vivo mono-colonization murine studies in *Escherichia coli* and *Klebsiella pneumoniae,* we highlight findings that introduced a paradigm shift by showing that nitrogen-scavenging mechanisms may be dispensable under certain conditions. Rather than refuting nitrogen limitation, we propose a re-evaluation of nutrient limitation in the gut, and a more nuanced model, with carbon and energy sources emerging as potential bottlenecks. This minireview emphasizes *Enterobacteriaceae* as a pathobiont model for investigating nitrogen metabolism and outlines key knowledge gaps to be addressed in future studies.

## Introduction

Nitrogen is an essential nutrient for the growth of all forms of life including bacteria, supporting core biosynthetic processes such as protein and nucleic acid synthesis (Kuypers et al. 2018; Reese et al. 2018; Elser et al. 2007). In the context of the mammalian gut microbiome, however, the availability and limitation of nitrogen remain subjects of debate.

Historically, the colon, the densest reservoir for the microbiome in the intestinal tract, has been considered to be nitrogen-limited, as the majority of the dietary amino acids (AAs) supplied through the host diet are absorbed in the small intestine before they reach the resident colonic microbiota(Reese et al. 2018; Borgström et al. 1957; Sender et al. 2016). The assumption that the gut microbiome utilizes host dietary protein has been supported by observations made in murine studies conducted under germ-free conditions. The introduction of gut bacteria resulted in an increase in host protein requirements by consumption of dietary nitrogen (Wostmann 1981).

Nitrogen limitation does not have a universally applied definition. Some infer the definition from the decrease in intracellular glutamine levels, however, the precise characteristics of this decrease have yet to be quantified (Heeswijk et al. 2013). Alternative sources define it as an environment, in which external nitrogen supplementation leads to an increase in primary production and therefore in biomass (Tamm and Schulze 1991). This perspective is supported by studies showing an increase in microbial biomass when dietary protein intake, and therefore nitrogen, was increased, promoting the claim that the gut is nitrogen-limited (Reese et al. 2018). This has been further supported by studies demonstrating extensive nitrogen-scavenging mechanisms within the gut microbiota, suggesting that bacteria are actively competing for nitrogen sources (Zimmer et al. 2000; Bender 2010). We will subsequently refer to the latter proposed definition as nitrogen limitation.

However, recent studies have challenged this notion, proposing instead that nitrogen may not be the predominant factor limiting the growth of the intestinal microbiome under all circumstances. Instead, other factors, such as carbon or energy sources, may impose stronger constraints on bacterial gut colonization (Hecht et al. 2024; Doranga and Conway 2024; Muramatsu and Winter 2024).

We will critically examine the evidence surrounding available nitrogen sources in the gut and the role of nitrogen-scavenging mechanisms in microbial colonization. We will explore alternative nitrogen acquisition pathways and the context of nitrogen utilization in the microbiome. Due to their clinical and scientific significance as opportunistic pathogens, we will be mainly focusing on *Enterobacteriaceae*, while acknowledging that nitrogen assimilation strategies in dominant anaerobic taxa of the gut microbiome remain underexplored. Although not the central focus, related mechanisms in other members of the gut microbiota will be addressed when relevant. By combining findings from classical and recent literature, we aim to provide a balanced perspective on the factors shaping microbial nitrogen metabolism in the intestinal tract.

## Potential nitrogen sources for the gut microbiome

**Fig. 1:**
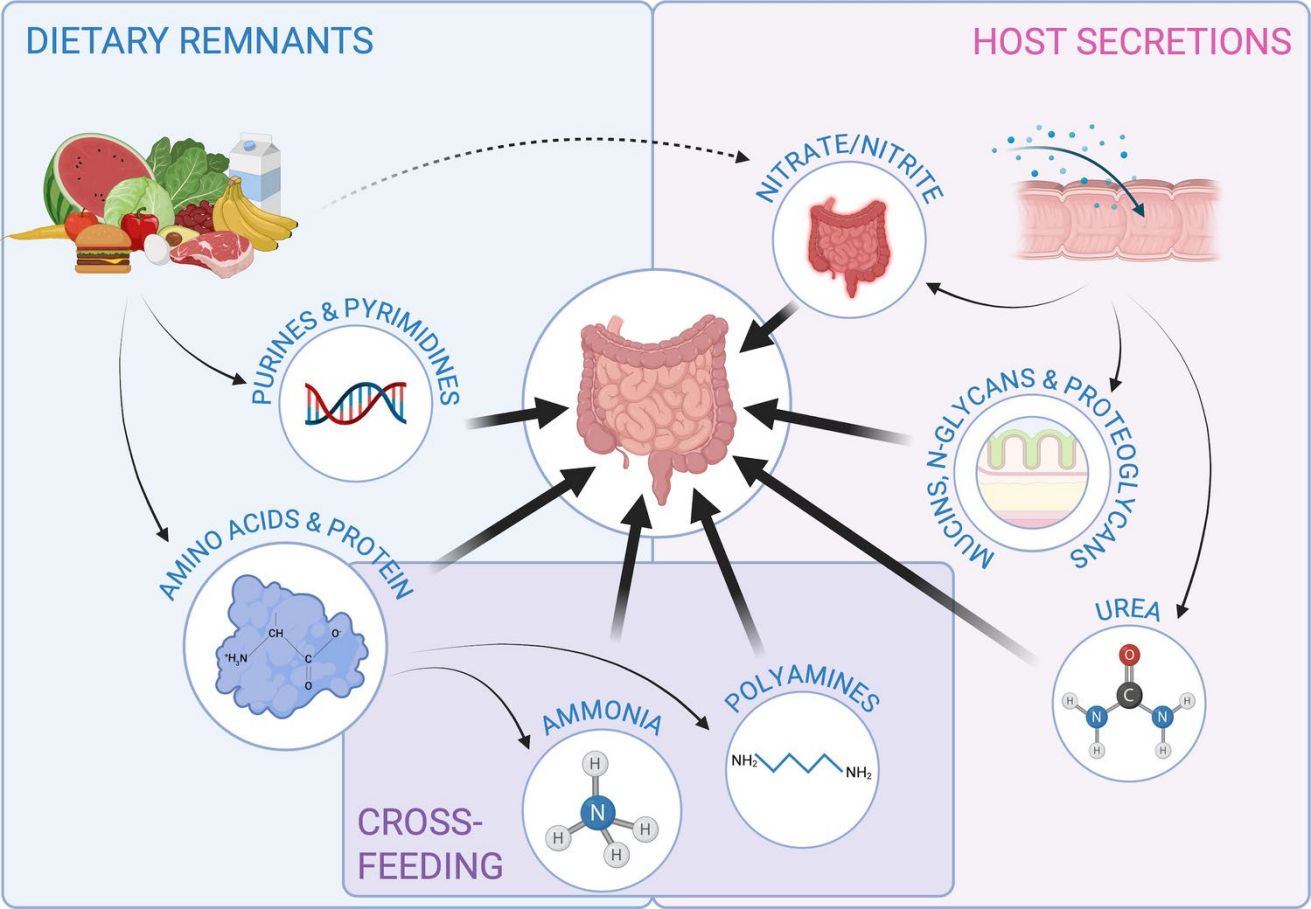
Synoptic graphical representation of the available nitrogen sources in the gut microbiome and interactions between the prescribed systems of dietary remnants, host secretions, and cross-feeding of nitrogen sources. Created with BioRender.com/txgstam

In terrestrial and aquatic ecosystems microbial growth is often dependent on the nitrogen cycle, in which elemental dinitrogen gas is transformed into bio-available forms of nitrogen by nitrogen-fixing bacteria and archaea (Kuypers et al. 2018). Most organisms are, however, dependent on the accessibility of bio-available nitrogen, which, in the gut, is provided through a wide array of nitrogen-containing compounds, derived from both exogenous sources, e.g. dietary residues, and host-derived secretions (mucins, sloughed intestinal epithelial cells, urea, and secretions from various digestive organs), as well as microbial interactions (Fig. 1) (Kuypers et al. 2018; Reese et al. 2018; Zeng et al. 2022; Fuller and Reeds 1998). Major contributors to the gut microbiome, such as Bacteroidetes, Firmicutes, Actinobacteria, or Proteobacteria, can utilize a great variety of compounds as sources of nitrogen (Doranga and Conway 2024; Khanna et al. 2014; Bergen and Wu 2009).

### Dietary remnants

Dietary AAs, dipeptides, and protein provide a substantial part of potential nitrogen sources (Doranga and Conway 2024; Bergen and Wu 2009). Peptides are degraded by either the microbiota or the epithelium and free AAs can be further metabolized (Bergen and Wu 2009). Some AAs can be utilized as both a source of nitrogen and carbon (Doranga and Conway 2024).

Purines and pyrimidines arise through the diet, and from host and microbial cell turnover and can be metabolized by the microbiome (Kasahara et al. 2023; Yang et al. 2024). Additionally, the end product of the purine metabolism in the host, uric acid, is excreted into the gut lumen and can be metabolized by the microbiota(Kasahara et al. 2023; Sorensen and Levinson 1975).

### Host secretions

Urea plays an important role in the nitrogen pool of the intestinal tract and enters the gut lumen passively, mainly deriving from the host’s metabolism, and can be hydrolyzed to supply ammonia via the enzyme urease (Zeng et al. 2022; Fuller and Reeds 1998; Bergen and Wu 2009; Suzuki et al. 1979). The resulting ammonia is either used as a source of nitrogen by the microbiome or reabsorbed by the host, reentering the urea cycle (Zeng et al. 2022; Bergen and Wu 2009; Moran and Jackson 1990).

Nitrate and nitrite are inorganic anions that can derive from the diet or the nitric oxide synthase pathways, which are involved in inflammatory processes (Nakamura 2023; Winter et al. 2013). They can be utilized as electron acceptors in anaerobic energy production by the microbiome and, thereby, result in the formation of ammonia (Nakamura 2023; Winter et al. 2013; Besson et al. 2022; Lewis and Gui 2023).

Mucins, as well as glycosaminoglycans (GAGs), are glycoproteins found as part of the glycocalyx covering the intestinal epithelium (Hua and Hasnain 2022). They can be connected to the epithelial cell surface or be secreted and consequently form a gel over the mucosa via the hydrophilic O-glycosylation of their protein core (Hua and Hasnain 2022). The intestinal mucin layer and the microbiome have a bidirectional relationship, as the composition of the gut microbiome is influenced by the repertoire of mucins, as they can be metabolized by some species, and, vice versa, the microbiome affects the composition of mucins through commensal mucolytic bacteria (Hua and Hasnain 2022).

N-glycans are part of cell membranes, both in the intestinal epithelium and as dietary components, and can be accessed by some members of the microbiome as a source of nitrogen (Stanley et al. 2022; Sastre et al. 2024). They are a result of N-glycosylation, through which oligosaccharides are attached to the side chain of asparagine in proteins (Stanley et al. 2022). Similarly, proteoglycans consist of a protein, to which GAGs are attached via various linkages, depending on the specific proteoglycan that can contain oligosaccharides, O-glycans, or even N-glycans themselves (Stanley et al. 2022; Silbert 1982; Merry et al. 2022). GAGs are further comprised of disaccharide chains, partly containing amino sugars (Merry et al. 2022). They are predominantly found in cell membranes, the extracellular matrix, or as storage forms and reach the gut microbiome through the host’s diet or tissue turnover (Merry et al. 2022; Dong et al. 2024; Cordeiro et al. 2023).

Multiple members of Bacteroidetes, as well as some Firmicutes, and Proteobacteria, encode for specific enzymes in so-called polysaccharide utilization loci (PULs) that gain them the ability to hydrolyze glycans found in the intestinal tract (Sastre et al. 2024; Dong et al. 2024; Cordeiro et al. 2023; Brockhausen et al. 2022). Even though the catabolism of glycans is mostly associated with carbon metabolism, the amino sugars, N-acetylgalactosamine and N-acetylglucosamine, that can be found as a foundational component of glycoproteins can be further metabolized (Stanley et al. 2022; Merry et al. 2022; BioCyc. 2025). The product of N-acetylglucosamine metabolism, for example, can be incorporated into the glycolysis for energy production, while simultaneously resulting in ammonia production as a source of nitrogen (BioCyc. 2025). The ability to access host secretions as sources of carbon and nitrogen is widely utilized by Bacteroidetes, and gives the bacteria an advantage in environments where nutrition from the host’s diet is limited (Holmes et al. 2017).

## Nitrogen scavenging and assimilation mechanisms in the gut microbiome

The intestinal microbiome uses various methods to assimilate available nitrogen sources. However, as some will be more easily accessible, most organisms have specific nitrogen sources that are preferably utilized, as well as variations in the extent to which different compounds will be employed as nitrogen sources (Heeswijk et al. 2013). Bacteria such as *Enterobacteriaceae*, specifically *E. coli*, *Klebsiella pneumoniae,* or *Salmonella enterica*, possess multi-layered systems that act to regulate nitrogen metabolism and consist of diverse control elements (Heeswijk et al. 2013).

Many of the mentioned metabolic pathways are mainly investigated in model organisms such as *E. coli*, yet the majority of the gut microbiota is primarily composed of anaerobes, mainly Firmicutes, and Bacteroidetes, which are often not included in these metabolic investigations.

### Core assimilation pathways

Ammonia is a leading source of nitrogen for the gut microbiome and facilitates the greatest growth as a nitrogen supplier in vitro (Heeswijk et al. 2013). The main pathways of ammonia assimilation in *E. coli* are described as the glutamate dehydrogenase (GDH) pathway, which is predominantly utilized when ammonia is abundant, and the glutamine synthetase (GS)-glutamate synthase (GOGAT) pathway, which is employed in low-ammonia, or nitrogen-limited, environments (Heeswijk et al. 2013; Yuan et al. 2009). The GS-GOGAT pathway costs the bacteria more energy in the form of adenosine triphosphate (ATP), but exhibits a higher affinity to the then-rare substrate of ammonia, and is therefore advantageous over the low-affinity GDH pathway in the case of nitrogen limitation (Heeswijk et al. 2013).

Even though the majority of the microbiome can synthesize proteinogenic AAs from other nitrogen sources via ammonia, readily available AAs from dietary protein are preferably utilized (Zeng et al. 2022). While non-essential AAs, which are more closely related to tricarboxylic acid (TCA) cycle derivatives, are partly synthesized by the microbiome, essential AAs are primarily derived from the host’s diet (Zeng et al. 2022). In opposition to ruminants, where AA production by the microbiome plays a substantial part in the AA supply for the host, the uptake of microbiome-produced AAs by non-ruminants, or monogastric hosts, is believed to be insignificant, as some studies report no detected isotopic labeled AAs, produced by the microbiota in the host’s circulation (Zeng et al. 2022; Fuller and Reeds 1998). Opposingly, other studies suggest that microbial protein can in fact be absorbed by the monogastric host, as tracer-labeled microbial proteins have been observed in the pool of free plasma AAs (Fuller and Reeds 1998; Lin et al. 2017). This implies that some members of the gut microbiome may have a preference for utilizing readily accessible nitrogen in the form of AAs before utilizing other nitrogen sources to biosynthesize the needed AAs.

Proteins from the host’s diet that reach the microbiome in the colon are hydrolyzed by proteases and peptidases, fragmenting the proteins into peptides and single AAs (Gibson et al. 1976; Diether and Willing 2019). However, the assimilation of single free AAs is not the most energetically profitable for the bacteria, and peptides are therefore preferably imported (Diether and Willing 2019). These AAs and peptides can be incorporated into microbial proteins, further metabolized, or used as an energy source through distinct pathways (Lin et al. 2017; Diether and Willing 2019; Neis et al. 2015). The assimilation of AAs for metabolism does not occur at the same rate for all available AAs. Overall, hydrophilic peptides and AAs are primarily metabolized, leading to serine, arginine, and aspartate being preferentially imported and primarily metabolized, while hydrophobic AAs, such as the aromatic tyrosine, tryptophan, and phenylalanine, are metabolized at a slower rate (Smith and Macfarlane 1998).

One pathway of AA catabolism is deamination, resulting in the formation of ammonia and a keto-acid, by removing the amino group from the AA, which can then be used as a source of nitrogen (Diether and Willing 2019; Krebs 1935; Rasmussen et al. 1988). Alternatively, AAs can be decarboxylated, which in turn produces biogenic amines, such as cadaverine from lysine decarboxylation, agmatine from arginine, or histamine from histidine, which may subsequently impact host physiology (Neis et al. 2015).

The pathway of fermentation, which mainly takes place in the more anaerobic cecum and colon, is conducted by various members of the microbiome, for example Firmicutes such as *Clostridium spp.*, *Actinomyces spp.*, or *Fusobacterium spp.,* as well as Bacteroidetes*,* or Proteobacteria (Lin et al. 2017; Diether and Willing 2019; Neis et al. 2015; Porter and Larsbrink 2022; Lin and Visek 1991). As a result of dietary fiber, as well as to a smaller extent AA, fermentation, short-chain fatty acids (SCFAs) are formed, which can subsequently be utilized by the host (Holmes et al. 2017; Smith and Macfarlane 1998; Krebs 1935; Rasmussen et al. 1988). The most abundant SCFA in the gut is acetate, which is produced by the fermentation of glycine, threonine, glutamate, or ornithine, with the following minor SCFAs being propionate, which is derived from threonine, as well as butyrate, which is a product of lysine, glutamate, and again threonine fermentation(Lin et al. 2017; Smith and Macfarlane 1998; Rasmussen et al. 1988; Macfarlane and Allison 1986). *Clostridium spp.* is known as the main fermenter of lysine and proline, while fermentation of aromatic AAs happens mostly through *Escherichia spp*. and their assimilation occurs at a slower rate compared to other AAs (Diether and Willing 2019; Smith and Macfarlane 1998). In addition to SCFAs, branched-chain fatty acids (BCFAs) are the result of branched-chain AA (valerate, isobutyrate, isovalerate) fermentation, which is mainly employed by *Bacteroides spp*. (Diether and Willing 2019; Neis et al. 2015; Rasmussen et al. 1988). The production of SCFAs and BCFAs from fermentation vice versa impacts the fermentation of AAs by decreasing the luminal pH and subsequently the rate of ammonia production from fermentation (Smith and Macfarlane 1998; Lin and Visek 1991).

These pathways are not exclusive to free AA metabolism, as AA pairs can be metabolized via the Stickland reaction, predominantly by *Clostridium spp.*, which consists of a coupled redox reaction. The reaction consists of one AA acting as the electron donor and being oxidized, while the other, as the electron acceptor, is being reduced (Barker 1954). This leads to the deamination of the electron donor AA, as well as the production of ammonia and SCFAs or BCFAs (Barker 1954).

Urease is an enzyme produced by various bacterial species, both commensal and pathogenic, and commonly found in the intestinal tract of animal hosts (Suzuki et al. 1979; McLean et al. 1988; Konieczna et al. 2012; Mobley et al. 2001). It catalyzes the hydrolysis of urea, producing carbamic acid, carbonic acid, and ammonia (Mobley et al. 1995). This reaction can have various effects on the bacteria and its environment but ultimately facilitates the accessibility of urea as an additional nitrogen source (Mobley et al. 1995; Rutherford 2014). Urease-positive organisms are part of the resident microbiome of the intestinal tract, one example being *K. pneumoniae *(Joseph et al. 2021; Ni et al. 2017). Their outgrowth, however, can be associated with dysbiosis (Ni et al. 2017).

Other than making urea accessible as an additional nitrogen source, urease can be utilized to neutralize acidic environments. One example of this mechanism is *Helicobacter pylori*. It is well-established that *H. pylori* is capable of utilizing urease to neutralize gastric acid through the production of the H^+^-acceptor ammonia (NH_3_) via the hydrolysis of urea(Konieczna et al. 2012; Mobley et al. 1995). Urease in *Staphylococcus aureus* is another example of the enzyme increasing acid tolerance, as its expression increases during weak acidic stress in the urinary tract and facilitates urinary tract infections with *S. aureus *(Zhou et al. 2019).

Facultative anaerobes, such as *E. coli*, can utilize nitrate or nitrite as an alternative electron acceptor to produce ATP in an anaerobic environment (Winter et al. 2013; Besson et al. 2022; Tiso and Schechter 2015). Despite this predominantly relating to carbon and energy metabolism, the process additionally produces ammonia (Besson et al. 2022; Tiso and Schechter 2015). This is important to consider as nitrate produced by the inducible nitric oxide synthase during inflammation can be advantageous for *E. coli,* and lead to an outgrowth of the bacterium (Winter et al. 2013). Obligate anaerobes of Bacteroidetes, such as *B. thetaiotaomicron*, or *Lactobacillus plantarum*, a Firmicute, were additionally observed to be able to utilize nitrate as an electron acceptor and simultaneously produce ammonia (Lewis and Gui 2023; Tiso and Schechter 2015).

### Nitrogen regulatory networks

In low-nitrogen environments, indicated mainly by low-glutamine levels, nitrogen-scavenging mechanisms come into play that can make additional nitrogen sources accessible to the bacteria (Bender 2010; Sánchez-Cañizares et al. 2020). Examples of these are the nitrogen regulatory (Ntr) system, the phosphotransferase system (PTS), or PULs that grant the organism access to alternative nitrogen sources (Zimmer et al. 2000; Bender 2010; Doranga and Conway 2024; Sastre et al. 2024; Dong et al. 2024; Cordeiro et al. 2023; Pflüger-Grau and Görke 2010). These systems often impact not only nitrogen metabolism but also carbon metabolism, leading to a precise interplay of both systems that maximizes the utilization of present resources (Bender 2010; Sánchez-Cañizares et al. 2020; Pflüger-Grau and Görke 2010). Products of these pathways can often be utilized by other members of the microbiota, adjacent to the producing organism (Culp and Goodman 2023; Goyal et al. 2021).

The Ntr system was discovered after experiments in 1973 revealed that nitrogen limitation would lead to changes in enzyme production in *Klebsiella spp.* and can be found in *Enterobacteriaceae*, such as *K. pneumoniae* or *E. coli *(Zimmer et al. 2000; Bender 2010; Doranga and Conway 2024; Prival et al. 1973). It relies on low cellular glutamine levels, which reflect the nitrogen limitation, for its activation and facilitates the utilization of alternative nitrogen sources by the bacteria (Bender 2010). When the cellular glutamine concentration decreases, *glnALG* is transcribed, which leads to the production of GS (*glnA*), NtrB (*glnL*), and NtrC (*glnG*) (Blauwkamp and Ninfa 2002). GS assimilates ammonia, via glutamine production, at a higher rate by utilizing ATP (Heeswijk et al. 2013). The transcription factor NtrC is phosphorylated by NtrB and thereby activated, which subsequently leads to the transcription of the *nac* gene (Bender 2010). Following this, the NAC protein regulates multiple pathways regarding nitrogen metabolism but has also been found to be involved in carbon metabolism, cell division, and the synthesis of macromolecules (Bender 2010). NtrC ~ P additionally regulates *glnALG*, a gene that encodes for NtrB and NtrC, leading to a positive autoregulatory loop (Blauwkamp and Ninfa 2002).

Examples of genes activated by NAC and the resulting metabolic pathways include *hut*, responsible for the degradation of histidine into ammonia, glutamate, and formamide, *ure*, involved in the hydrolysis of urea into ammonia and carbon dioxide, *cod*, leading to the degradation of cytosine into ammonia and uracil, *dad*, which leads to the degradation of alanine into ammonia and pyruvate, and *oppA*, which encodes an oligopeptide transporter (Bender 2010). NAC is not only responsible for the activation but also the repression of genes, such as *gdhA*, which induces the formation of glutamate from ammonia and α-ketoglutarate via GDH, and *gltB*, responsible for the synthesis of glutamate from glutamine and α-ketoglutarate via GOGAT (Bender 2010). These genes are mainly expressed when ammonia is abundant and NAC is not synthesized (Bender 2010).

The Ntr System and the regulation of metabolic pathways via the NAC protein have additionally been observed in *E. coli*. However, in this species, the scope of the Ntr systems is suggested to be limited to the metabolism of nitrogen (Zimmer et al. 2000; Bender 2010). Here the genes activated by the Ntr system mainly encode transporters for the scavenged nitrogen compounds (Zimmer et al. 2000). Some genes that are regulated by NAC in *K. pneumoniae* were also found in *E. coli*, such as *cod*, or *opp*. Meanwhile, other genes, like *pot*, and *ydc*, encode for a putrescine/spermidine transporter, which are polyamines that can be metabolized as a nitrogen source, *ast*, involved in the arginine catabolism, *argT*, encoding for a basic AA transporter, *ddp*, resulting in the synthesis of the transporter for the dipeptide D-alanine-D-alanine, as well as a corresponding dipeptidase, and *cycA*, which encodes for the D-alanine-transporter, seem to be more specific to *E. coli *(Zimmer et al. 2000; Hua and Hasnain 2022).

In other *Enterobacteriaceae* whose genetic code is heavily studied, such as *S. enterica*, the same regulation has not been observed, despite the existing Ntr system, as it lacks the *nac* gene (Bender 2010).

Ammonia is an easily accessible nitrogen source for the majority of the microbiome and leads to the greatest growth as a sole nitrogen source in vitro (Heeswijk et al. 2013). It can either diffuse passively into the cell or be transported by AmtB in cases of limited ammonia supply (Heeswijk et al. 2013; Merrick et al. 2006). The activity of AmtB is coupled to the protein GlnK, produced via the gene *glnK*, which regulates the phosphorylation of NtrC and subsequently the expression of Ntr genes (Blauwkamp and Ninfa 2002; Merrick et al. 2006). GlnK is modified during nitrogen excess and binds AmtB, which subsequently inhibits excess ammonia assimilation (Merrick et al. 2006). If nitrogen is scarce, the modifications are removed from GlnK, disinhibiting AmtB and increasing ammonia uptake (Merrick et al. 2006).

The PTS was first identified in *E. coli* as a mechanism of carbohydrate transport (Pflüger-Grau and Görke 2010). In the following years, a second type of the system has been discovered, which is involved in the regulation of nitrogen metabolism, called PTS^Ntr^ (Pflüger-Grau and Görke 2010). In comparison to the carbohydrate metabolism controlling PTS, which is omnipresent in the microbiota, the PTS^Ntr^ is mainly found in gram-negative bacteria, especially Proteobacteria(Pflüger-Grau and Görke 2010). Multiple species present in the gut microbiome have been found to possess PTS^Ntr^, such as *K. pneumoniae*, and *E. coli*, as well as other bacteria, such as some *Pseudomonas spp. *(Pflüger-Grau and Görke 2010).

The system consists of multiple enzymes, namely enzyme I (EI^Ntr^), and the histidine protein Npr, that transfer phosphate groups from phosphoenolpyruvate onto a membrane-bound transporter, enzyme II A (EIIA^Ntr^) (Pflüger-Grau and Görke 2010). The enzymes involved in the PTS^Ntr^ are homologous to the ones found in the carbohydrate PTS (Pflüger-Grau and Görke 2010). The system is suspected to be able to sense nitrogen availability through changes in phosphorylation mediated by glutamate and α-ketoglutarate, subsequently regulating the nitrogen metabolism by activating ATP-binding cassette transporters as well as interacting with the homeostasis of potassium (Sánchez-Cañizares et al. 2020).

The PTS^Ntr^ is thought to contribute to a connection between the nitrogen and carbon metabolism, independent of the Ntr system, gaining the bacteria vital adaptability to changes in both the carbon and nitrogen supply (Sánchez-Cañizares et al. 2020; Pflüger-Grau and Görke 2010). If there is a surplus of available nitrogen, the carbon metabolism might be increased by upregulating the TCA cycle activity to increase overall metabolism, while during nitrogen limitation, the excess carbon, not utilized in the metabolism, can be stored (Sánchez-Cañizares et al. 2020). The PTS^Ntr^, therefore, interacts with the homeostasis of nitrogen and carbon in the metabolism by regulating the uptake of amino acids, as well as the TCA cycle (Sánchez-Cañizares et al. 2020).

### Microbial cross-feeding

The products resulting from the aforementioned metabolic pathways (ammonia, free AAs, peptides, biogenic amines) can not only be consumed by the bacteria that they originated from but can be used by other members of the resident microbiota through cross-feeding. The preferred sources of nitrogen differ between phyla (Zeng et al. 2022). Ammonia, for example, is an often-employed nitrogen source in vitro and is available in the intestinal tract from host secretions through the urea cycle and through cross-feeding of the microbiome, a result of metabolic reactions, such as the hydrolysis of urea or the deamination of AAs (Zeng et al. 2022; Bergen and Wu 2009). Polyamines, namely spermine, spermidine, and putrescine, are biogenic amines that arise from the metabolism of arginine, ornithine, and lysine by the microbiome in the mammalian gut and can be utilized through cross-feeding (Sagar et al. 2021). *Bacteroides* frequently utilize host-secreted proteins, such as mucins and glycans, and therefore have a competitive advantage in phases of limited dietary nitrogen influx, while Firmicutes obtain the majority of the utilized nitrogen from the host’s diet (Zeng et al. 2022).

While many studies have shown cross-feedings between members of the gut microbiome in vitro, there is less evidence for the application of this process in vivo, regarding nitrogen sources (Culp and Goodman 2023).

Due to the complexity and limitation in research opportunities on cross-feeding interaction of the gut microbiome in vivo, computational methods have been recently employed to predict possible interplay from metagenomic and metabolomic data (Goyal et al. 2021). Noteworthy predictions are the cross-feeding of glutamate, alanine, and glutamine, as well as metabolites from AA catabolism, such as BCFAs, with a main focus on isovalerate, SCFAs, specifically butyrate, and polyamines, such as putrescine (Goyal et al. 2021). Additionally, a recent study modeled the murine gut microbiome in a synthetic community, made of 12 members of the five main phyla of the gut (Firmicutes, Bacteroidetes, Proteobacteria, as well as Actinobacteria, and Verrucomicrobia) (Pérez Escriva et al. 2022). They observed the main interaction of cross-feeding of nitrogen compounds in the microbiome to involve arginine, which was mainly produced by Bacteroidetes and consumed by Firmicutes (Pérez Escriva et al. 2022). Other amino acids, suggested to be involved in cross-feeding, were cysteine, which is produced by different members of Firmicutes and predominantly consumed by the Firmicute *Clostridium clostridioforme,* as well as histidine, which originates from Bacteroidetes to be consumed by Firmicutes (Pérez Escriva et al. 2022). Another relevant observation that was made was the cross-feeding of the purine metabolites xanthine and hypoxanthine, wherein hypoxanthine is produced by *Bifidobacterium* and some Firmicutes and can subsequently be consumed by Bacteroidetes, as well as *C. clostridioforme *(Pérez Escriva et al. 2022). *C. clostridioforme* is further the main consumer of xanthine (Pérez Escriva et al. 2022).

## Shifts in the understanding of nitrogen limitation in the gut microbiome

Nitrogen scavenging mechanisms have traditionally been considered essential for microbial colonization of the large intestine, an environment traditionally viewed as nitrogen-limited due to early absorption of dietary AAs in the small intestine (Reese et al. 2018; Borgström et al. 1957; Sender et al. 2016). As a result, bacteria in the colon have been thought to rely predominantly on host-secreted and microbially derived nitrogen sources (Reese et al. 2018; Zeng et al. 2022; Fuller and Reeds 1998). This is further supported by observations of a two-fold increase in the carbon-to-nitrogen ratio in the lumen from the proximal small intestine to the colon, indicating a relative scarcity of nitrogen in distal gut regions (Reese et al. 2018).

Recent experimental findings have begun to challenge the classical paradigm. Investigations into the distribution of AA transporters along the intestinal tract have shown that the colon retains the capacity to absorb protein, including branched-chain, aromatic, and neutral AAs (Bröer and Fairweather 2018). Moreover, different microbial taxa appear to exhibit distinct preferences for ratios of host-secreted to dietary nitrogen (Holmes et al. 2017). Bacteroidetes are known to utilize host-secreted resources and tend to dominate when dietary sources of nitrogen become scarce, whereas Firmicutes often prefer a nutrient supply from dietary sources (Holmes et al. 2017). Notably, contrary to previous assumptions, the relative ratio of dietary protein to host-secreted protein increases along the intestinal tract, with the highest ratio of dietary to secreted protein in the colon, which was observed in a study utilizing isotopically labeled nitrogen in both conventionally-raised and germ-free mice (Reese et al. 2018). This increase in the dietary-to-secreted protein ratio may be due to the fact the gut microbiome in the large intestine, and partly also in the small intestine, preferentially utilized readily available AAs from dietary sources to conserve energy that would otherwise be needed for AA synthesis (Hecht et al. 2024; Zeng et al. 2022). Experimental studies utilizing *K. pneumoniae* mutants deficient in either urease or NtrC, encoded by *glnG*, provide further nuance (Hecht et al. 2024). While both mutants exhibit impaired growth under nitrogen-limited conditions in vitro, these disadvantages do not affect colonization fitness in vivo, even under a low-protein host diet supplying reduced dietary nitrogen sources (Hecht et al. 2024). This would suggest that under certain conditions, such as in mono-colonized murine models, urease and the Ntr system as a nitrogen assimilation mechanism may be dispensable for *K. pneumoniae* colonization. Nevertheless, their role may vary in more complex nutrient or community contexts. Another study observed a similar effect in *E. coli* mutants deficient in *glnG* that exhibited a growth defect only in direct competition with the NtrC-competent WT, but not in monocolonization (Doranga and Conway 2024). These observations would suggest that the Ntr system, activated through NtrC, is involved in the colonization fitness of *E. coli *in vitro*,* as well as being advantageous in direct competition in vivo but can be dispensable for colonization of the intestinal tract in a murine model. Transcriptomic analysis revealed that NtrC-dependent genes were downregulated during gut colonization, even in WT *E. coli*, resembling the gene expression profile of the *glnG* mutant (Doranga and Conway 2024). Furthermore, pathways involved in AA biosynthesis were downregulated during gut colonization, and biosynthesis of the high-affinity ammonium transporter AmtB was not upregulated, both suggestive of sufficient nitrogen availability during colonization (Doranga and Conway 2024). In germ-free mice colonized with a urease-deficient strain of *K. pneumoniae*, a substantial increase of fecal ammonia after the introduction of the bacteria suggests that an excess of nitrogen is available and at least partially discarded (Hecht et al. 2024). Additionally, ex vivo experiments showed that the supplementation of ammonia to sterilized intestinal contents did not improve *K. pneumoniae* growth, further suggesting that ammonia was not growth-limiting under those conditions (Hecht et al. 2024).

**Fig. 2:**
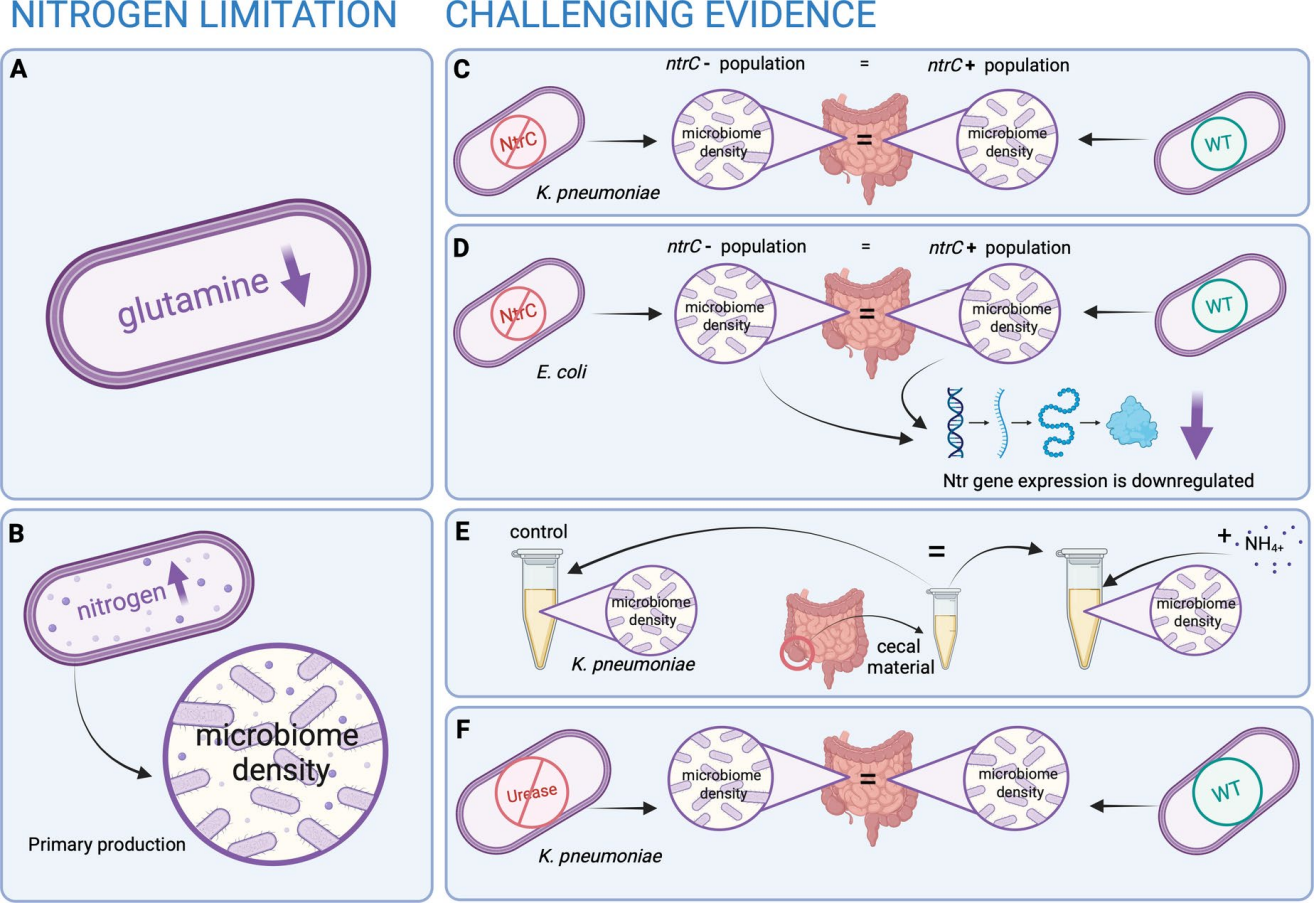
Synopsis of the challenges against the hypothesis of nitrogen limitation in the monogastric intestinal tract. Definition of nitrogen limitation as **A** low cellular glutamine levels (Heeswijk et al. 2013) or **B** an increase in primary production in case of nitrogen supplementation (Tamm and Schulze 1991). Challenges to the hypothesis of the monogastric gut being nitrogen-limited: **C** deletion of *ntrC* in *K. pneumoniae* does not lead to colonization deficits (Hecht et al. 2024); **D** expression of Ntr genes is downregulated in both WT *E. coli* and mutants lacking *ntrC during* colonization (Doranga and Conway 2024); **E** colonization of *K. pneumoniae* in ex vivo cecal material does not increase with supplementation of ammonia (Hecht et al. 2024); **F** deletion of the urease operon in *K. pneumoniae* does not lead to colonization deficits (Hecht et al. 2024). Created with BioRender.com/ej7wlw5

Taken together, these findings challenge the assumption that the mammalian intestinal tract is universally nitrogen-limited and suggest a more context-based approach (Fig. 2). If nitrogen limitation is defined as a state in which low intracellular glutamine concentrations lead to compensatory scavenging pathways or where nitrogen supplementation increases microbial mass, then these studies suggest that these conditions are not consistently met in vivo. Key elements of the Ntr system (*glnALG*, *nac*), as well as GlnK, which subsequently disinhibits AmtB to increase the assimilation of available ammonia, were not upregulated during colonization, and deletion of *glnG*, impeding the activation of the Ntr system, did not impair fitness of either *K. pneumoniae* or *E. coli* in monocolonization (Hecht et al. 2024; Doranga and Conway 2024; Blauwkamp and Ninfa 2002; Merrick et al. 2006).

Instead of nitrogen, some studies point towards carbon as a more critical growth-limiting nutrient in the gut microbiome (Hecht et al. 2024; Muramatsu and Winter 2024). Isotope tracing experiments have shown that the availability of specific carbon sources had a distinct influence on the biosynthesis of AAs and microbial biomass more directly than nitrogen supply (Hecht et al. 2024; Muramatsu and Winter 2024; Deng et al. 2021). A study investigating the interaction between nitrogen and carbon intake also observed that nitrogen limitation stands in close relation to carbon intake, yet nitrogen limitation appears to be the dominant and driving factor in community assembly(Holmes et al. 2017).

Glucose supplementing significantly enhanced growth in cecal contents, while ammonia supplementation had no comparable effect (Hecht et al. 2024). This suggests that ammonia was not limiting under these circumstances but does not exclude the possibility that other forms of nitrogen or their assimilation may constrain growth in increasingly complex environments.

However, it is important to interpret these findings within their methodological context. Colonization levels were assed via colony-forming units per g of stool, which is not an unrestricted representation of the mucosal colonization in the intestinal tract and site-specific sampling might give a better estimate of the gut colonization (Hecht et al. 2024). Utilizing an antibiotic regimen and a polyethylene glycol flush as a pretreatment may impact the nutrient composition of the utilized cecal extract (Hecht et al. 2024).

Additionally, urease and the Ntr systems are not solely responsible for nitrogen acquisition and alternative routes for nitrogen assimilation (AA transporters, PTS^Ntr^, PULs, etc.) remain active and may compensate under different environmental conditions.

The observed findings may indicate a trend, suggesting that nitrogen limitation in the intestinal tract is not as pressing as long believed, yet they do not provide conclusive evidence against the hypothesis of nitrogen limitation and additional research investigating additional nitrogen assimilation systems will be necessary. Additional investigations of the complex relationship between the gut microbiome and the available nutrients in the intestinal tract will be needed to clarify their interaction, as it is possible that specific circumstances, such as inflammation of the intestinal tract, may increase the advantageous effects of nitrogen assimilation mechanisms.

## Data Availability

No datasets were generated or analysed during the current study.

## Abbreviations

ATP: Adenosine triphosphate
AA: Amino acid
*B. thetaiotaomicron*: *Bacteroides thetaiotaomicron*
BCFA: Branched-chain fatty acid
*C. clostridioforme*: *Clostridium clostridioforme*
*E. coli*: *Escherichia coli*
GAG: Glycosaminoglycan
GDH: Glutamate dehydrogenase
GOGAT: Glutamate synthase
GS: Glutamate synthetase
*H. pylori*: *Helicobacter pylori*
*K. pneumoniae*: *Klebsiella pneumoniae*
Ntr: Nitrogen regulatory system
PTS: Phosphotransferase system
PTS^Ntr^: Phosphotransferase system regarding nitrogen metabolism
PUL: Polysaccharide utilization loci
SCFA: Short-chain fatty acid
*S. enterica*: *Salmonella enterica*
*S. aureus*: *Staphylococcus aureus*
*spp.*: *Species*
TCA: Tricarboxylic acid
WT: Wild type
